# Exploring men’s cancer journeys in Norway: a comprehensive survey on diet, supplements, and use of complementary and alternative therapies

**DOI:** 10.1186/s12906-025-04748-7

**Published:** 2025-02-24

**Authors:** Agnete E. Kristoffersen, Kiwumulo Nakandi, Arne Johan Norheim, Mona Bjelland, Jorunn V. Nilsen, Eran Ben-Arye

**Affiliations:** 1 National Research Center in Complementary and Alternative Medicine (NAFKAM), Department of Community Medicine, UiT The Arctic University of Norway, Tromsø, Norway; 2 The Norwegian Cancer Society, Oslo, Norway; 3 Integrative Oncology Program, The Oncology Service, Lin, Zebulun, and Carmel Medical Centers, Clalit Health Services, Haifa, Israel; 4 Rappaport Faculty of Medicine, Technion-Israel Institute of Technology, Haifa, Israel

**Keywords:** Cancer, CAM, Diet, Men, Oncology

## Abstract

**Background:**

Each year, over 20,000 men are diagnosed with cancer in Norway, and approximately 150,000 men who have previously been diagnosed with cancer are currently alive. Many of these cancer survivors encounter a range of challenges, including fatigue, sexual dysfunction, urinary issues, and pain, all of which can significantly impact their quality of life. Consequently, a substantial number of men seek support beyond conventional healthcare. This study aims to investigate the motivations behind the use of Complementary and Alternative Medicine (CAM) and dietary changes/supplements in men with cancer in Norway, and further explore their communication with healthcare providers, self-reported effects and adverse effects, and the sources of information they rely on regarding these practices.

**Method:**

In collaboration with the Norwegian Cancer Society (NCS), we conducted an online cross-sectional study involving participants of their user panel who have current or past experiences with cancer (*n* = 706), of whom 218 identified as men. The study was conducted during the autumn of 2021, employing a modified cancer-specific version of the International Questionnaire to Measure Use of Complementary and Alternative Medicine (I-CAM-Q). A total of 153 men agreed to participate, yielding a response rate of 70%.

**Results:**

A large proportion of the respondents used CAM (62%), dietary supplements (65%), and/or adjusted their diet (81%) to boost their immune systems and increase their quality of life. The dietary adjustments involved eating more fruits, vegetables, fish, and whole grains. Many participants also used relaxation techniques and visited CAM providers to enhance quality of life. Most participants reported better health outcomes as a result of these interventions. The Internet and healthcare professionals were the main source of information, although many did not disclose their dietary changes and CAM therapy use with healthcare professionals.

**Conclusion:**

By leveraging these insights, healthcare providers, policymakers, and researchers can collectively work towards a more holistic and patient-centred approach to cancer care, ultimately improving the overall well-being and quality of life for male cancer survivors.

## Background

More than 20,000 men are diagnosed with cancer in Norway each year with a median age at diagnosis of 70 years. By the end of 2023, approximately 150,000 men who had previously been diagnosed with cancer were living in Norway. Prostate cancer is the most common cancer site with 5258 new cases in 2023 followed by lung cancer (1696 cases) and colon cancer (1665 cases) [1]. Although prostate cancer has reached 5-years survival rates above 95%, and that 77.1% of men in general survive their cancer at least five years, cancer is the main cause of death in Norway responsible for the death of 5810 men in 2023 [2].

The 5-year relative survival rate among men has, however increased from 43.7 to 77.1% during the last 20 years. This led to more and more men living with late effects of cancer and cancer treatment toxicities such as fatigue, sexual problems, urinary tract problems, and pain [3, 4]. Sexual problems, reported in up to 85% of prostate cancer patients [5], can result in a loss of sexual intimacy and shame in addition to depression, frustration, disappointment, and lower general life happiness [5]. Given the high prevalence of cancer survival in men and the burden of cancer- and treatment-related concerns, there is a pressing need to explore avenues for improving patients’ quality of life (QoL) during survivorship inside as well as outside conventional health care.

An aspect that has gained attention in this context is the role of diet for cancer patients [6]. Despite the evident interest among patients for dietary guidance [7, 8], its accessibility remains limited in Norway, particularly in rural districts [9].

Global dietary guidelines for cancer patients commonly emphasize dietary modifications such as increasing fibre intake, consuming more fruits and vegetables, and reducing the consumption of meat and sugary foods [10–15]. However, Norwegian male cancer patients seem to have low adherence to dietary recommendations (4.6%) [16] although the majority make dietary changes after being diagnosed with cancer (81.3%), mainly increased intake of fruit and vegetables, fish, whole grain, and reduced intake of sugar [17]. International studies show, however that men are less likely to alter their nutritional intake after a cancer diagnosis compared with women, and are more likely to make food choices based on taste preference rather than health-related factors [18]. Beliefs about healthy eating are likely to originate from societal- and gendered-norms, with men being less health conscious than women. They might therefore be less likely to alter their dietary choices and to follow nutritional guidelines [16, 18–20].

Reasons for dietary changes in patients following cancer diagnosis include preventing recurrence, supporting therapy and health, and managing treatment effects [7]. In addition to dietary changes, a significant number of men turn to the use of dietary supplements after being diagnosed with cancer (with prevalence ranging from 2 to 73% across different European studies) [17, 21]. Dietary supplements, which consist of concentrated nutrients, are popular among male cancer survivors in Norway, with 65.6% using them. The most commonly used supplement is Vitamin D, followed by Omega 3, multivitamins, cod-liver oil, and vitamin C [17]. While enhancing well-being is a common reason, caution is advised due to potential interactions with cancer treatment, mainly with chemotherapy. Open communication between patients and healthcare professionals regarding dietary changes and supplement use is recommended in international as well as in Norwegian studies [22, 23].

In parallel to dietary changes, international studies show that men also seek use of Complementary and Alternative Medicine (CAM), with the expectations to increase the body’s ability to fight cancer, to improve physical and emotional well-being [24, 25], to improve their QoL and to strengthen their body and the immune system [24].

CAM covers medicinal products and practices that are not part of conventional medicine [26], mainly offered outside the public health care system [27]. In Norway, visits to CAM providers, use of natural remedies (including herbs), and self-help practices represent what people broadly define as CAM [28]. The CAM modalities most commonly used by men with cancer are natural remedies (49.3%), followed by self-help practices (40.6%) and visits to CAM providers (20.3%) [29].

Although CAM is commonly used during the course of cancer, men use CAM to a much lower degree than women [29, 30]. While there is rich data on CAM use in populations including both women and men in Norway, the dominance of women among CAM users results in limited data on CAM use in male cancer patients.

The present study was designed to map the motivation behind the use of CAM and dietary changes/supplements in men with cancer in Norway, and further explore communication with health care providers, self-reported effects and adverse effects, and sources of information.

## Methods

The Norwegian Cancer Society (NCS) is one of the largest patient organizations in Norway, comprising 128,000 members affected by cancer, of whom 34% are male. The NCS has established a dedicated user panel [31] consisting of cancer patients, relatives and bereaved. Some of the participants in the user panel are members of the NCS, but membership is not a requirement for participation in the panel. The purpose of the user panel is to leverage the unique knowledge and experiences of cancer patients, their relatives, and the bereaved regarding patient pathways and interactions with health and welfare services. By participating in the user panel, members and others can provide valuable feedback and influence the development of new activities and services. Participants of the user panel may respond to surveys up to ten times a year. Since 2016, the user panel has been an active part of the NCS’s efforts to enhance cancer care. Together with NCS an online cross-sectional web-based study was conducted among male and female participants of the NCS’ user panel. The study was carried out in the autumn of 2021 using a modified, cancer-specific version of the International Questionnaire to Measure Use of Complementary and Alternative Medicine (I-CAM-Q) [32].

### Participants

The NCS’s user panel encompasses 906 participants, of whom 706 either presently suffer from cancer or have a documented history of cancer. Among this population, 218 individuals identify as men. Study participants were approached by recruitment strategies for panel participation involving utilization of the NCS’s official website, engagement through various social media platforms, and participation in diverse social gatherings and events.

All eligible participants of the NCS’s user panel, aged 18 years or older with a current or prior diagnosis of cancer were invited to partake in the survey. Participants of the user panel who categorized as relatives of individuals with a history of cancer, were excluded from participation.

In this paper, we excluded female participants from the analyses to specifically focus on dietary adjustments and the use of supplements and CAM modalities among male cancer survivors. Consequently, the remainder of the methods section will detail the recruitment process for male participants.

### Recruitment and data collection

**Fig. 1:**
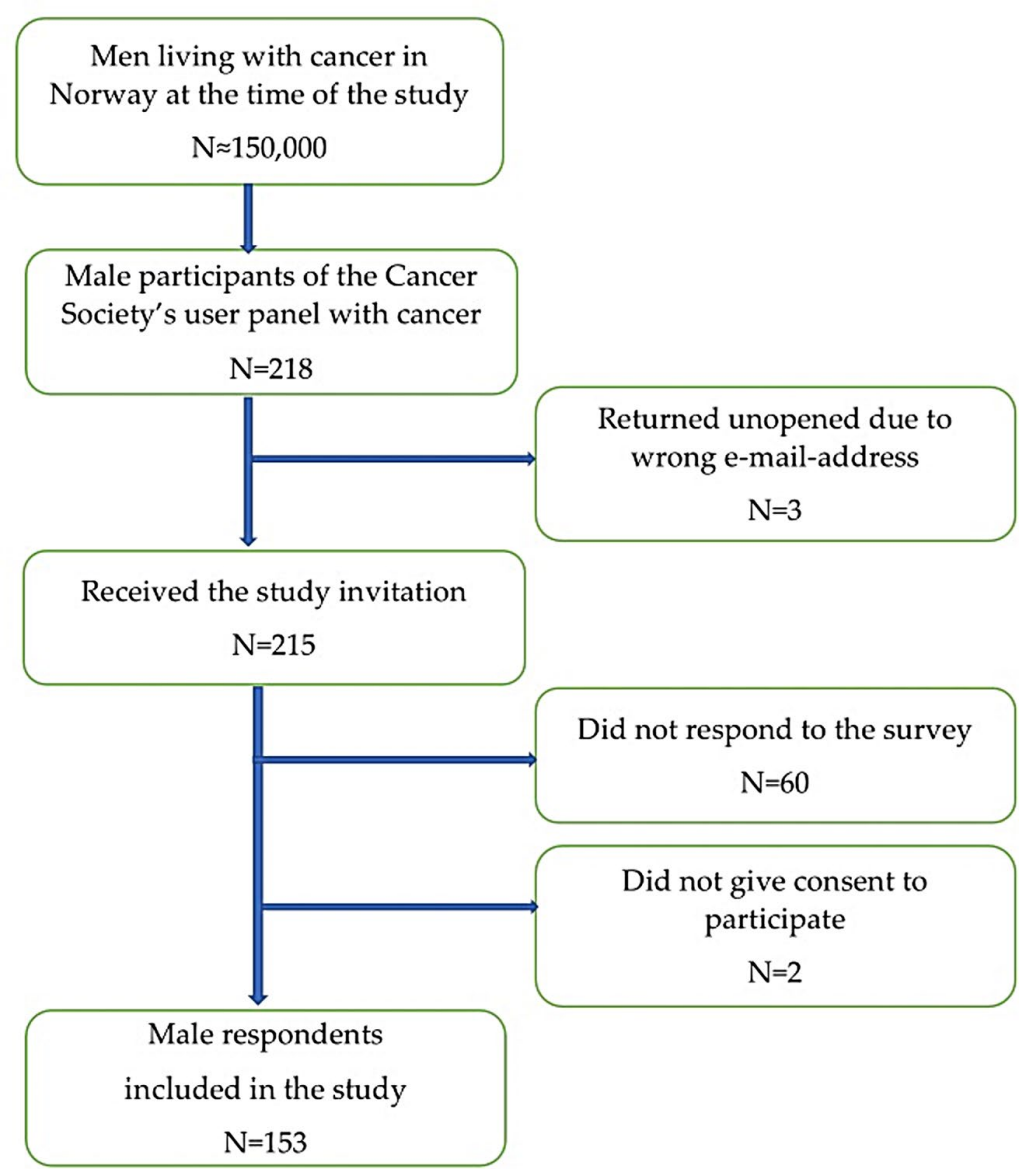
Flow chart of the included participants

All participants in the panel who met the inclusion criteria, including the 218 individuals identifying as male, received an email from the NCS with a link to the survey. The first page of the survey was an information letter where participants had to tick “agree to participate” to fill in the main survey. The survey was distributed online only. A total of three e-mails were returned as undeliverable leading to 215 men receiving the invitation. A total of 155 men responded. However, two did not give their consent to participate and were excluded from the study. Consequently, 153 of the 215 men agreed to participate resulting in a response rate of 70.2% (Fig. 1).

### Measures

The I-CAM-Q was developed based on the National Research Center in Complementary and Alternative Medicine (NAFKAM) model for classifying CAM use [32]. It encompasses visits to CAM providers, the use of natural remedies, self-help practices, dietary supplements, special diets, physical activity, and spiritual practices. For this study, additional questions regarding dietary modifications were included. Socio-demographic data, such as income and education, were collected, while data on age, gender, and cancer diagnosis were pre-collected by the NCS when participants joined the user panel.

For each modality used, participants were asked to specify their *reasons for use*, with response options including: (1) *To treat/slow down the cancer or prevent the cancer from spreading*; (2) *Treat adverse effects / late and long-term effects of the cancer or cancer treatment*; (3) *Strengthen the body / immune system*; (4) *Increase quality of life*,* coping*,* relaxation or well-being*; (5) *Other reasons*. Participants were also asked about *adverse effects* related to the interventions and rated their severity using the following options: (1) *Yes*,* serious*; (2) *Yes*,* moderate*; (3) *Yes*,* mild*; (4) *No*; (6) *Do not know*.

After addressing each intervention individually, participants were asked to evaluate the interventions collectively. This group-level assessment included CAM providers, natural remedies, self-help practices, dietary modifications, adherence to special diets, and the intake of vitamins and minerals. Participants assessed the *perceived effectiveness* of these interventions with options: (1) *Experienced improvement*; (2) *No change*; (3) *Worsened*; (4) *Do not know*. Additionally, participants indicated their *sources of information*: (1) *Internet/media*; (2) *Health* professionals (doctor/nurse, etc.); (3) *CAM provider*; (4) *Friends/family*, etc.; (5) *Other;* (6) *Do not remember/do not know*; (7) *Did not receive/seek information*. They also reported whether they discussed these interventions or modifications with healthcare providers, choosing from: (1) *General Practitioner*; (2) *Oncologist*; (3) *Nurse*; (4) *Other health professionals (nutritionist*,* etc.)*; (5) *CAM provider*; (6) *None of these*; (7) *Do not remember/do not know*.

### Measures of personal characteristics

Age was collected through an open-ended question and subsequently evaluated as both a continuous and categorical variable. After consolidating the data, participants were grouped into the following categories: *33–50 years*, *51–64 years*, and *65–82 years*, reflecting the age ranges of the study participants.

Educational levels were initially collected under four distinct categories and subsequently consolidated into three: (1) *Primary School*, (2) *Secondary School*, and (3) *College/University*.

Household yearly income data were collected and classified into the following categories: *Less than NOK 400*,*000* (EUR 34,000, designated as *low income*); *NOK 400*,*000 to NOK 799*,*000* (EUR 34,000 to 68,000 categorized as *medium income*); and *NOK 800*,*000 or above* (EUR 68,000 or above classified as *high income*). Additionally, participants were provided with the option to withhold their income information.

Additional personal characteristics encompassed gender (female, male) and residential location, which were consolidated into the Norwegian regions of South-East, South, West, Central (Trøndelag), and North of Norway.

### Statistics/ power calculation

To achieve adequate study power, a minimum sample size of *n* = 384 was required to represent the male Norwegian cancer population of 150,000, based on a 5% margin of error, a 95% confidence level, and 50% heterogeneity. Additionally, a minimum sample of *n* = 140 was needed to represent the male population of the NCS’s user panel of 218 [33]. Descriptive statistics were carried out using cross-tabulation and frequency analyses. For between-group analyses, Pearson chi-square tests and Fisher exact tests were used for categorical variables while independent sample t-tests were used for continuous variables. Significance level is reported at *p* < 0.05 and *p* < 0.10 levels, and the analyses were conducted using SPSS V.29.0 for Windows.

## Results

### Basic characteristics of the participants

The survey participants consisted of men aged between 33 and 82 years of age, with a mean age of 63 years. A significant portion held university degrees (58.7%) and boasted high income levels. Geographically, the majority of respondents resided in the southeastern region of Norway (48.4%). Male genital (prostate (28.8%), testicular (5.2%), or penis (0.7%)) cancer emerged as the predominant cancer type (34%), mostly in the post-cancer treatment phase. A substantial number (79%) of respondents reported enduring, long-term effects stemming from their cancer and its treatment. These effects encompassed QoL-related concerns like fatigue (48%), sexual difficulties (35%), and sleep disorders (24%, as indicated in Table 1).

**Table 1:** Basic characteristics of the participants and associations for dietary changes/supplements and CAM

	Total		Made dietary changes (modifications and/or special diets)			Used dietary supplements			Used CAM (CAM providers, natural remedies and/or self-help practices)		
	%	*n* = 153	%^1^	*n* = 127	*p*-value	%^2^	*n* = 106	*p*-value	%^3^	*n* = 89	*p*-value
**Age**					0.619^			0.323			0.184*
33–50 years	13.3	19	84.2	16		78.9	15		63.2	12	
51–64 years	37.1	53	81.1	43		66.0	35		52.8	28	
65–82 years	49.7	71	87.3	62		77.1	54		69.0	49	
Mean age years (SD)	62.92 (10.408)		63.02 (10.284)		0.772**	63.04 (10.610)		0.695**	63.67 (10.476)		0.265**
**Education**					0.048^			0.601^			0.376^
Primary school	9.1	13	69.2	9		61.5	8		46.2	6	
Secondary school	32.2	46	93.5	43		75.6	34		60.9	28	
College/ University	58.7	84	82.1	69		73.8	62		65.5	55	
Household income					0.665^			0.061^			0.200^
Low (Less than EUR 34,000)	10.5	15	80.0	12		73.3	11		66.7	10	
Middle (EUR 34,000 to 68,000)	33.6	48	87.5	42		68.8	33		50.0	24	
High (EUR 68,000 or more)	50.3	72	84.7	61		80.3	57		69.4	50	
No reply	5.6	8	75.0	6		37.5	3		62.5	5	
**Household** ^**1**^											
Live alone	16.3	25	96.0	24	0.086^	80.0	20	0.469*	60.0	15	0.799*
Live with a partner	75.2	115	82.6	95	0.205*	72.8	83	0.670*	63.5	73	0.535*
Live with own children	11.8	18	83.3	15	1.000^	55.6	10	0.085^	66.7	12	0.678*
Other	1.3	2	100	2	1.000^	100	2	0.609	100	2	0.527^
**Place of residence (region)**					0.521^			0.748^			0.523^
South-East	48.4	74	80.8	59		70.4	50		66.7	48	
South	4.6	7	71.4	5		85.7	6		42.9	3	
West	29.4	45	88.6	39		78.6	33		61.0	25	
Central (Trøndelag)	9.2	14	92.3	12		76.9	10		66.7	8	
North	8.5	13	92.3	12		63.6	7		45.5	5	
**Cancer site** ^**1**^											
Male genitalia	34.0	52	84.3	43	0.931*	66.7	32	0.181*	58.3	28	0.494*
Gastrointestinal	20.3	31	87.1	27	0.786^	76.7	23	0.670*	54.8	17	0.337*
Lymphoma	13.7	21	76.2	16	0.323^	85.7	18	0.173*	65.0	13	0.784*
Head and neck	8.5	13	84.6	11	1.000^	83.3	10	0.733^	58.3	7	0.765^
Malignant melanoma	5.2	8	75.0	6	0.354^	62.5	5	0.435^	50.00	4	0.476^
Lung	4.6	7	85.7	6	1.000^	85.7	6	0.676^	85.7	6	0.254^
Leukemia	2.6	4	75.0	3	0.490^	75.0	3	1.000^	75.0	3	1.000^
Bone marrow	2.6	4	100	4	1.000^	25.0	1	0.056^	100	4	0.297^
Sarcoma	1.3	2	100	2	1.000^	100	2	1.000^	50.0	1	1.000^
Other cancer sites	21.6	33	90.3	28	0.412^	76.7	23	0.670*	62.1	18	0.983*
Mean number of cancer sites	1.16 (SD 0.451)										
**In active cancer treatment**					0.347*			0.444*			0.177*
Yes	30.7	47	88.9	40		77.8	35		70.5	31	
No	69.3	106	82.9	87		71.7	71		58.6	58	
**Late and long-term effects**					0.021^			0.168*			0.889*
**No**	16.8	24	70.0	21		75.5	85		63.3	19	
**Yes**:	79.0	113	88.5	100		81.7	85		61.9	70	
Fatigue	47.7	73	89.0	65	0.148*	76.7	56	0.392*	65.8	45	0.376*
Sexual problems	34.6	53	94.3	50	0.015*	77.4	41	0.436*	56.6	30	0.286*
Sleep disorder	23.5	36	94.4	34	0.062*	77.8	28	0.512*	72.2	26	0.153*
Decreased muscle strength and mobility	22.2	34	94.1	32	0.082*	81.8	27	0.223*	70.6	24	0.250*
Urinary tract problems	22.2	34	100	34	0.005*	82.4	28	0.186*	58.8	20	0.638*
Pain	21.6	33	93.9	31	0.094*	75.8	25	0.750*	66.7	22	0.550*
Nerve damage (polyneuropathy)	20.9	32	84.4	27	1.000^	75.0	24	0.840*	62.5	20	0.972*
Hot flashes	16.3	25	84.0	21	1.000^	88.0	22	0.073*	56.0	14	0.479*
Anxiety or depression	16.3	25	100	25	0.015^	88.0	22	0.073*	80.0	20	0.044*
Gained weight	13.7	21	100	21	0.045^	90.5	19	0.058*	7.4	15	0.347*
Cognitive challenges	13.1	20	95.0	19	0.314^	85.0	17	0.280	70.0	14	0.440*
Diarrhea	13.1	20	95.0	19	0.314^	85.0	17	0.213*	70.0	14	0.440*
Mouth/tooth-problems and reduced taste	12.4	19	84.2	16	1.000^	73.7	14	0.994*	47.4	9	0.151*
Constipation	11.8	18	88.9	16	1.000^	83.3	15	0.402^	55.6	10	0.532*
Lymphedema	8.5	13	76.9	10	0.422^	75.0	9	1.000^	53.8	7	0.557^
Reduced fertility	8.5	13	100	13	0.220^	69.2	9	0.745^	46.2	6	0.239^
Weight loss	8.5	13	92.3	12	0.693^	76.9	10	1.000^	61.5	8	1.000^
Heart- and lung problems	7.0	10	80.0	8	0.651^	70.0	7	0.724^	70.0	7	0.743^
Other late and long-term effects	15.0	23	91.3	21	0.531^	82.6	19	0.285*	78.3	18	0.084*

* Pearson chi square test; ** Independent sample t-test; ^Fisher exact test; ^1^ The percentages in this column represent the percentage in each group who made dietary changes; ^2^ The percentages in this column represent the percentage in each group who used dietary supplements; ^3^ The percentages in this column represent the percentage in each group who used CAM

### Modifications to existing diet and adoption of special diets

Modifying an existing diet typically entails making incremental adjustments to the foods and nutrients consumed while maintaining the overall structure of the diet. This process may involve the addition or removal of specific foods. In contrast, adherence to specialized diets often necessitates a more substantial transformation of dietary habits. This could include adopting a new eating pattern, such as a vegetarian diet, or completely eliminating certain food groups. The dietary adjustments (including modifications to existing diet and adoption of special diets) were more frequently undertaken by men with a secondary schooling background (93.5%, *p* = 0.048), and among those who experienced long-term effects from their cancer or cancer treatment (88.5%, *p* = 0.021). Notably, these adjustments were strongly associated with managing conditions such as anxiety or depression (100%, *p* = 0.015), urinary tract issues (100%, *p* = 0.005), weight gain (100%, *p* = 0.045), sleep disorders (94.4%, *p* = 0.062), sexual difficulties (94.3%, *p* = 0.015), reduced muscle strength and mobility (94.1%, *p* = 0.082) as well as pain (93.9%, *p* = 0.094, Table 1).

### Modifying an existing diet

Most of the men (81.3%) modified their existing diet in relation to their cancer disease, mainly to strengthen the body and immune system (68%) and to improve their QoL, coping, relaxation, or well-being (64.8%, Table 2). Some also modified their diet to treat cancer/ prevent it from spreading (19%) or to treat side effects or late effects of cancer(treatment)(26%) while 46% had other reasons for modifying their diet. The most common modifications made were increased intake of fruit and vegetables (54%), fish (50.7%), whole grain (45.3%), and reduced intake of sugar (44.7%, Table 2). Out of the participants who made modifications to existing diet, 44% reported experiencing improvements following modification of their diet (Table 2).

**Table 2:** Use of dietary supplements and change of diet; reason(s), benefits, and adverse effects

			Reason(s) for use*													
	Total use^		To treat cancer/ prevent it from spreading		To treat side effects or late effects of cancer(treatment)		To strengthen the body / immune system		To increase QoL, coping, relaxation or well-being		Other reason		Improvement^1^		Adverse effects (severe, moderate or mild)	
	%	*n*	%	**n**	%	**n**	%	**n**	%	**n**	%	**n**	%	**n**	%	*n*
**Modification of existing diet**	**81.3**	**122**	**18.9**	**23**	**26.2**	**32**	**68.0**	**83**	**64.8**	**79**	**45.9**	**56**	**43.8**	**53**	**13.9**	**17**
Increased intake of fruit and vegetables	54.0	81	9.9	8	7.4	6	76.5	62	45.7	37	16.0	13	-	-	11.1	9
Increased intake of fish	50.7	76	7.9	6	7.9	6	61.8	47	52.6	40	17.1	13	-	-	5.3	4
Increased intake of whole grains	45.3	68	10.3	7	13.2	9	58.8	40	48.5	33	19.1	13	-	-	11.8	8
Reduced intake of sugar	44.7	67	17.9	12	23.9	16	46.3	31	50.7	34	23.9	16	-	-	11.9	8
Reduced intake of alcohol	36.7	55	9.1	5	18.2	10	40.0	22	52.7	29	25.5	14	-	-	1.8	1
Reduced intake of dairy products	23.3	35	14.3	5	22.9	8	20.0	7	48.6	17	40.0	14	-	-	14.3	5
Reduced intake of carbohydrates	22.0	33	21.2	7	21.2	7	27.3	9	57.6	19	30.3	10	-	-	18.2	6
Reduced intake of meat	19.3	29	17.2	5	17.2	5	31.0	9	51.7	15	31.0	9	-	-	10.3	3
**Special diets**	**41.9**	**62**	**16.1**	**10**	**12.9**	**8**	**67.7**	**42**	**48.4**	**30**	**24.2**	**15**	**48.4**	**30**	**6.5**	4
Nutrient dense	24.3	36	5.6	2	5.6	2	66.7	24	44.4	16	11.1	4	-	-	8.3	3
Ecological	14.2	21	19.0	4	14.3	3	61.9	13	71.4	15	28.6	6	-	-	0.0	0
Low carb	10.8	16	37.5	6	25.0	4	37.5	6	43.8	7	37.5	6	-	-	6.3	1
Fasting	5.4	8	12.5	1	12.5	1	62.5	5	62.5	5	12.5	1	-	-	0.0	0
Vegetarian	2.0	3	33.3	1	0.0	0	33.3	1	66.7	2	0.0	0	-	-	0.0	0
Ketogenic	2.7	4	75.0	3	25.0	1	50.0	2	25.0	1	0.0	0	-	-	0.0	0
Juice only (carrot, beetroot, apricot etc.)	3.0	3	66.7	2	33.3	1	100	3	100	3	0.0	0	-	-	33.3	1
Budwig	1.4	2	100	2	0.0	0	50.0	1	0.0	0	0.0	0	-	-	0.0	0
**Dietary supplements**	**64.6**	95	**12.6**	**12**	**29.5**	**28**	**97.9**	**93**	**33.7**	**32**	**13.7**	**13**	**46.3**	**44**	**4.2**	4
Vitamin D	41.5	61	4.9	3	24.6	15	82.0	50	18.0	11	6.6	4	-	-	-	-
Omega 3 fatty acid	31.3	45	6.7	3	15.6	7	88.9	40	31.1	14	2.2	1	-	-	-	-
Multivitamins	28.6	42	14.3	6	26.2	11	85.7	36	26.2	11	4.8	2	-	-	-	-
Cod-liver oil	26.4	38	7.9	3	13.2	5	97.4	37	21.1	8	5.3	2	-	-	-	-
Vitamin C	25.2	37	10.8	4	16.2	6	81.1	30	16.2	6	2.7	1	-	-	-	-
Vitamin B	21.1	31	9.7	3	22.6	7	77.4	24	19.4	6	3.2	1	-	-	-	-
Antioxidant	17.7	26	19.2	5	19.2	5	96.2	25	15.4	4	0.0	0	-	-	-	-
Zink	10.2	15	13.3	2	26.7	4	66.7	10	20.0	3	20.0	3	-	-	-	-
Vitamin E	6.8	10	10.0	1	30.0	3	90.0	9	20.0	2	0.0	0	-	-	-	-
Vitamin A	5.4	8	25.0	2	25.0	2	75.0	6	25.0	2	25.0	2	-	-	-	-
Vitamin K	4.1	6	16.7	1	50.0	3	83.3	5	0.0	0	0.0	0	-	-	-	-
Iodine	2.7	4	25.0	1	50.0	2	75.0	3	25.0	1	25.0	1	-	-	-	-
Selenium	2.0	3	33.3	1	66.7	2	100	3	33.3	1	0.0	0	-	-	-	-
Other vitamins and minerals	20.4	30	-	-	-	-	-	-	-	-	-	-	-	-	-	-

*Multiple choice; ^The total number of men using the different diets and supplements are presented in previous papers [17, 29], and are added here as a background to the information presented in the rest of the Table ^1^ For improvement, the information was collected for each group of intervention rather than for intervention separately: - data not collected

**Table 3:** Self-reported effect, information and disclosure of dietary changes, supplements and CAM use

	Dietary modifications		Special diets		Dietary supplements		Natural remedies		Self-help practices		Provider based CAM therapies	
	%	*n* = 122*	%	*n* = 62*	%	*n* = 95*	%	*n* = 52*	%	*n* = 58*	%	*n* = 29*
**Self-reported effect**												
Better	43.4	53	48.4	30	33.7	32	32.7	17	72.4	42	86.2	25
No change	37.7	46	38.7	24	41.1	39	44.2	23	10.3	6	10.3	3
Worse	0.8	1	0.0	0	0.0	0	0.0	0	0.0	0	0.0	0
Don’t know	17.2	21	11.3	7	24.2	23	23.1	12	17.2	10	3.4	1
Information**												
Internet / media	36.9	45	45.2	28	32.6	31	51.9	27	36.2	21	24.1	7
Healthcare professionals	36.1	44	37.1	23	46.3	44	15.4	8	34.5	20	41.4	12
CAM providers	0.8	1	1.6	1	2.1	2	5.8	3	1.7	1	3.4	1
Friends, family	29.5	36	40.3	25	22.1	21	42.3	22	34.5	20	41.4	12
Other	14.8	18	17.7	11	9.5	9	13.5	7	12.1	7	20.7	6
Do not remember	6.6	8	3.2	2	2.1	2	3.8	2	8.6	5	3.4	1
Did not seek/receive	18.0	22	11.3	7	7.4	7	11.5	6	20.7	12	3.4	1
Disclosure**												
Family physician	18.9	23	17.7	11	36.8	35	11.5	6	29.3	17	48.3	14
Oncologist	20.5	25	22.6	14	17.9	17	15.4	8	29.3	17	24.1	7
Nurse	9.0	11	8.1	5	5.3	5	7.7	4	19.0	11	6.9	2
CAM provider	0.0	0	0.0	0	6.3	6	9.6	5	12.1	7	17.2	5
Other health care providers	21.3	26	17.7	11	0.0	0	3.8	2	1.7	1	3.4	1
None of these	52.5	64	51.6	32	40.0	38	61.5	32	46.6	27	37.9	11
Do not remember	3.3	4	0.0	0	6.3	6	3.8	2	5.2	3	3.4	1

*Due to missing responses the sum of the numbers does not always add up to the total number; **Multiple choice

### Special diets

The majority of participants who adopted special diets following their cancer diagnosis did so to enhance their body’s strength and immune system (67.7%) or to improve quality of life, coping, relaxation, or overall well-being (48.4%). A smaller portion modified their diet to treat cancer or prevent its spread (16%), or to address side effects or late effects of cancer and its treatment (13%). Additionally, 24% of participants had other reasons for following a special diet. Nearly half of the participants (48.4%) adopting special diets found them beneficial.

### Dietary supplements

Dietary supplements were commonly used by participants (64.6%), primarily to strengthen the body and immune system (97.9%). Some participants used supplements to address side effects or late effects of cancer and its treatment (29.5%), and to enhance quality of life, coping, relaxation, or overall well-being (33.7%). A few used dietary supplements to treat cancer or prevent its spread (12.6%), or for other reasons (13.7%). Among those using dietary supplements, 46.3% reported experiencing improvements due to their use.

Men with higher incomes showed a notable preference for dietary supplements (80.3%, *p* = 0.061) enduring, long-term effects such as weight gain (90.5%, *p* = 0.058) as well as managing concerns of anxiety and depression (88%, *p* = 0.073) and hot flashes (88%, *p* = 0.073). Among men with bone marrow cancer, the utilization of dietary supplements was comparatively lower in comparison to those facing other cancer diagnoses (25%, *p* = 0.056, Table 1).

### Complementary and alternative medicine (CAM)

This study examined the use of CAM modalities, including natural remedies, self-help practices, and visits to CAM providers (for detailed modalities, see Table 3). The use of CAM, whether provider-based or self-administered was positively associated with anxiety and depression (80%, *p* = 0.044), with no significant associations found with other factors.

### Natural remedies

Natural remedies were used less frequently (36%) than dietary supplements. However, like dietary supplements, they were primarily utilized to strengthen the body and immune system (90.4%) and to enhance quality of life, coping, relaxation, or overall well-being (42.3%). A notable proportion of participants also used natural remedies to treat cancer or prevent its spread (26.9%), as well as to address side effects or late effects of cancer and its treatment (23.1%). Additionally, 32.7% of participants reported experiencing improvements in their health or symptoms due to use of natural remedies.

**Table 4:** Use of CAM modalities; reason(s), benefits, and adverse effects

			Reason(s) for use***													
	Total use^		To treat cancer or prevent it from spreading		To treat side effects or late effects of cancer/ cancer treatment		To strengthen the body / immune system		To increase QoL, coping, relaxation or well-being		Other reason		Improvement^1^		Adverse effects (severe, moderate or mild)	
	%	n	**%**	**n**	**%**	**n**	**%**	**n**	**%**	**n**	**%**	**n**	**%**	**n**	%	n
**Natural remedies**	36.1	52	26.9	14	23.1	12	90.4	47	42.3	22	11.5	6	32.7	17	**1.9**	1
Garlic	18.1	26	23.1	6	11.5	3	84.6	22	38.5	10	15.4	4	-	-	3.8	1
Blueberries / blueberry extract	13.2	19	15.8	3	10.5	2	94.7	18	15.8	3	5.3	1	-	-	0.0	0
Ginger	11.8	17	17.6	3	17.6	3	82.4	14	41.2	7	5.9	1	-	-	0.0	0
Green tea	11.1	16	12.5	2	12.5	2	75.0	12	50.0	8	0.0	0	-	-	0.0	0
Turmeric / curcumin	8.3	12	41.7	5	25.0	3	83.3	10	41.7	5	0.0	0	-	-	0.0	0
Aloe Vera	4.2	6	0.0	0	33.3	2	50.0	3	16.7	1	16.7	1	-	-	0.0	0
Cannabis	1.4	2	100	2	0.0	0	50.0	1	0.0	0	0.0	0	-	-	0.0	0
Chaga	0.7	1	100	1	0.0	0	0.0	0	0.0	0	0.0	0	-	-	0.0	0
Echinacea	0.7	1	0.0	0	0.0	0	0.0	0	100	1	0.0	0	-	-	0.0	0
Q10	0.7	1	0.0	0	0.0	0	0.0	0	100	1	0.0	0	-	-	0.0	0
Ginseng	0.7	1	0.0	0	0.0	0	100	1	0.0	0	0.0	0	-	-	0.0	0
Medical mushrooms (Reishi, Maitake, Shitake)	0.7	1	100	1	0.0	0	100	1	0.0	0	0.0	0	-	-	0.0	0
Noni-juice	0.7	1	0.0	0	100	1	100	1	0.0	0	0.0	0	-	-	0.0	0
Birch sap	0.7	1	0.0	0	0.0	0	100	1	0.0	0	0.0	0	-	-	0.0	0
Other natural remedies**	3.5	5	-	-	-	-	-	-	-	-	-	-	-	-	-	-
**Self-help practices**	**40.6**	58	**6.9**	4	**20.7**	12	**43.1**	25	**96.6**	56	**1.7**	1	72.4	42	**12.1**	7
Relaxation	34.3	49	6.1	3	18.4	9	40.8	20	93.9	46	0.0	0	-	-	12.2	6
Meditation/mindfulness	10.5	15	6.7	1	26.7	4	33.3	5	93.3	14	0.0	0	-	-	0.0	0
Yoga	7.0	10	10.0	1	20.0	2	70.0	7	100	10	10.0	1	-	-	0.0	0
Music therapy	6.3	9	0.0	0	11.1	1	0.0	0	100	9	0.0	0	-	-	0.0	0
Visualisation	2.8	4	0.0	0	25.0	1	0.0	0	100	4	0.0	0	-	-	25.0	1
Tai chi / qi gong	1.4	2	0.0	0	0.0	0	100	2	100	2	0.0	0	-	-	50.0	1
Other self-help practices**	22.4	32	-	-	-	-	-	-	-	-	-	-	-	-	-	-
Consultations with CAM providers*	**20.3**	29	**3.4**	**1**	**41.4**	**12**	**17.2**	**5**	**69.0**	**20**	**10.3**	**3**	86.2	25	**10.3**	**3**
Psychotherapy**	16.8	24	0.0	0	20.8	5	4.2	1	91.7	22	0.0	0	-	-	4.2	1
Massage/aromatherapy	9.8	14	0.0	0	28.6	4	14.3	2	85.7	12	0.0	0	-	-	7.1	1
Acupuncture	6.3	9	0.0	0	66.7	6	22.2	2	22.2	2	0.0	0	-	-	11.1	1
Naprapathy	4.9	7	0.0	0	57.1	4	28.6	2	42.9	3	42.9	3	-	-	28.6	2
Healing	2.8	4	25.0	1	25.0	1	0.0	0	75.0	3	0.0	0	-	-	0.0	0
Osteopathy	0.7	1	0.0	0	0.0	0	0.0	0	100	1	0.0	0	-	-	0.0	0
Coaching	0.7	1	0.0	0	0.0	0	0.0	0	100	1	0.0	0	-	-	0.0	0
Other provider based CAM therapies*	11.2	16	-	-	-	-	-	-	-	-	-	-	-	-	-	-
Total use of CAM*	**62.2**	**89**	**18.0**	**16**	**32.6**	**29**	**67.4**	**60**	**77.5**	**69**	**11.2**	**10**	-	-	**10.1**	**9**

*Natural remedies, self-help practices and consultations with CAM provider; ** Not included in total CAM use due to uncertainty of this being CAM; ***Multiple choice; ^The total number of men using the different CAM therapies are presented in a previous paper [29], and are added here as a background to the numbers presented in the rest of the table; ^1^ For improvement, the information was collected for each group of intervention rather than for intervention separately: - data not collected

### Self-help practices

A significant portion of participants (40.6%) adopted self-help practices, primarily to enhance their quality of life, implement effective coping strategies, and promote relaxation and overall well-being (96.6%). Additionally, 41.3% of participants used these practices to strengthen their body and immune system. A smaller group (20.7%) engaged in self-help practices to address side effects or late effects of cancer and its treatment, while only a few (6.9%) utilized them to treat cancer or prevent its spread. Notably, the majority of men who engaged in self-help practices (72.4%) reported finding them beneficial (Table 3).

### Provider-based CAM therapies

Only one in five men visited a CAM provider in relation to their cancer, with the majority (69%) doing so to improve their quality of life. Acupuncture and naprapathy were, however primarily used to address side effects or late effects of cancer and its treatment (66.7% and 57.1%, respectively). Most men who visited CAM providers reported experiencing improvements as a result of the treatment (86.2%, Table 3).

### Risk factors

While one participant experienced a worsening of his condition following dietary changes, most men reported either improvements or no significant changes in their health status after implementing dietary modifications, supplements, and CAM approaches. Nevertheless, it is important to acknowledge the occurrence of adverse effects.

The majority of adverse effects were documented following dietary changes (13.9%), with nine cases classified as serious, ten as moderate, and 23 as mild. Adverse effects were primarily reported after increased intake of fruits and vegetables (*n* = 9), whole grains (*n* = 8), and reduced sugar consumption (*n* = 8). Additional reports of adverse effects followed increased intake of fish (*n* = 4) and decreased intake of carbohydrates (*n* = 6), dairy products (*n* = 5), meat (*n* = 3), and alcohol (*n* = 1).

Furthermore, a subset of individuals reported adverse effects after engaging in self-help practices (12.1%), with five cases categorized as mild and three as moderate. These effects were mainly reported after relaxation practices (*n* = 6), with single reports following tai chi/qi gong and other self-help practices. Visits to CAM providers also resulted in adverse effects in some instances (10.3%), with three cases considered as mild and two as moderate. Adverse effects were mainly reported after naprapathy (*n* = 2), with single reports following visits to massage therapists and acupuncturists. Fewer individuals reported adverse effects associated with the use of special diets (6.5%), dietary supplements (4.2%), and natural remedies (1.9%). Regarding special diets, adverse effects were reported after adopting a nutrient-dense diet (*n* = 3), a low-carb diet (*n* = 1), and a juice diet (*n* = 1). Adverse effects from natural remedies were reported following the use of garlic (*n* = 1). Adverse effects from dietary supplements were reported at a group level, with four men experiencing issues after using multivitamins, vitamin A, vitamin B, vitamin C, vitamin D, vitamin E, and other unspecified vitamins and minerals.

### Sources of information and communication patterns

As shown in Table 4, respondents utilized diverse sources to gather information regarding nutrition and CAM. The Internet emerged as the predominant source of information, with the highest reliance for natural remedies (51.9%), specialized diets (42.5%), dietary modifications (36.9%), self-help practices (34.4%), and provider-based CAM therapies (24.1%). Alongside the Internet, healthcare professionals were also a significant source of information, prominently sought after for dietary supplements (41.5%) and provider-based CAM therapies (41.2%). Additionally, respondents turned to healthcare professionals for guidance on self-help practices (38.3%), specialized diets (37.1%), and dietary modifications (36.1%). Input from family and friends played a substantial role, particularly in the context of natural remedies (42.3%) but also encompassing provider-based CAM therapies (41.2%), specialized diets (40.3%), dietary modifications (29.5%), and self-help practices (28.5%).

Many of the men did not discuss their dietary changes and CAM therapy use with healthcare professionals, especially use of natural remedies (61.5%), dietary modifications (52.5%), and special diets (52.5%). When these topics were discussed, the conversations primarily took place with oncologists (15.4%, 20.5%, and 22.6% respectively) or family physicians (11.5%, 18.9%, and 17.7% respectively). Dietary modifications and special diets were also occasionally discussed with other health professionals, such as nutritionists etc. (21.3% and 17.7% respectively). The most frequently discussed topic with healthcare providers was visits to CAM practitioners, where family physicians were consulted 48.3% of the time, followed by oncologists at 24.1% (Table 4).

## Discussion

### Main findings

A large proportion made dietary changes and used herbs and supplements to boost their immune systems and overall well-being. These changes often involved eating more fruits, vegetables, fish, and whole grains. Many also used relaxation techniques and visited CAM providers to improve their QoL. Most participants reported better health outcomes as a result of these interventions. The Internet was the main source of information for natural remedies and special diets, while healthcare professionals were consulted for guidance on dietary supplements and provider-based CAM therapies. Many individuals did not discuss these interventions with healthcare professionals, particularly when it came to natural remedies, dietary modifications, and special diets. When discussions did take place, family physicians were most frequently consulted, especially regarding provider-based CAM therapies, although conversations with oncologists also occurred.

### Other studies

The adoption of dietary changes and the use of herbal and non-herbal dietary supplements among cancer patients is well-documented in the medical literature [21, 34–38]. The present study provides a unique perspective by thoroughly investigating the motivations for using these interventions, the perceived benefits and risks, and the sources from which respondents obtained information about the modalities they employed. Additionally, it examines the extent to which these interventions were discussed with healthcare providers.

Though the use of some dietary changes and supplements is not unique to the Norwegian context (e.g., reducing sugar, the use of ginger and turmeric), some of these dietary-supplements use may be specifically related to Norway (e.g., eating fish, cod-liver oil) and to the patients’ health-belief model.

As early as 1997, Veierød and colleagues from the university of Oslo, explored prospectively a cohort of more than 50,000 men and women attending Norwegian health screening and reported significant lower lung cancer risk for cod liver oil supplement [39]. Somewhat later, in 2013, Torfadóttir et al. found lower risk of advanced prostate cancer among Icelandic men consuming fish liver oil in later life [40]. While some men in the present study used cod liver oil with the intention of curing cancer or preventing its spread, the majority used it to strengthen the body and support the immune system.

Although cod liver oil is traditionally used in Norway, many individuals opt for Omega-3 fatty acid capsules and vitamin D supplements instead, primarily due to the distinct taste of cod liver oil. Like cod liver oil, these supplements are mainly used to strengthen the body and support the immune system following a cancer diagnosis. This is also found in male cancer patients in the US, France, and Korea [41–43], although used less frequently. As Norway is Europe’s largest fishing nation and the 9th largest in the world [44], fish and fish products have traditionally played an important role both as food and health prevention and treatment, especially in the coastal areas. Fishing and hobby angling are common hobbies among men, contributing to higher fish consumption in subgroups of the population [45]. Fishing is one of many forms of food procurement in Norway with free fishing in the sea and some lakes, and fresh, wild fish available in the supermarkets at a reasonable price. This may be one of the reasons why men wanting to improve their diet after a cancer diagnosis choose to increase their fish intake. In addition, fishing can be seen as more than just a means of obtaining fish. It can also be regarded as a lifestyle ‘intervention’ and even as a mind-body experience considering the intimate impact of being connected with nature [46].

This study does not answer to what extent male patients with cancer adopt dietary changes and supplements use based on their affiliation with Norwegian traditions. Fish and blueberries are typical to the Norwegian cuisine, and conciderated as ‘nourishing’ and ‘healthy’ alongside cod liver oil. On the other hand, nutrition among men, might under normal circumstances be challenged by low intake of fruit [47] and increased intake of sugar [48]. The increased intake of fruit and reduction in the consumption of sugar might bring these men closer to the national diet recommendations [49] rather than a level in accordance with these recommendations. This is suspected as only 4.6% of Norwegian males with cancer adhere to the national recommendations for fruit and vegetables in the diet [50].

The findings of dietary changes made most frequently to increase QoL, successfully by only 44% of the respondents, is in accordance with a systematic review concluding that dietary changes have been shown to partly affect QoL in cancer patients [51].

In the present study, the relatively high utilization of self-help practices contrasts with the fact that only 16.8% of men had undergone psychotherapy. This observation may suggest an inclination of men to cope with stress and anxiety non-verbally. Contrary to the to the stereotype of the stoic male who “shoot, don’t talk” as depicted in the movie The Good, the Bad, and the Ugly, a prospective study involving informal caregivers in a neuro-intensive care unit found that male caregivers may benefit from strategies focused on increasing intimate care [52].

The present study, which suggests that men often solve their mental challenges with physical approaches, is supported by a Canadian study examining the effects of a mindfulness meditation-based stress reduction program on mood and symptoms of stress in cancer outpatients. Carlson et al. concluded that this modality is beneficial in both males and females with a wide variety of cancer diagnoses and illness stages [53]. Furthermore, a meta-analysis of mindfulness and meditative movement interventions for men living with cancer found evidence, albeit with a small effect in favour of these interventions, for improving psychosocial outcomes in male cancer survivors [54].

The present study also sheds light on the suboptimal doctor-patient communication as less than 50% of the respondents disclosed their dietary modifications to healthcare professionals. Although dietary recommendations are essential components in oncology clinical guidelines [14, 55], the fact that only 15.4% of the respondents reported to have received guidance from healthcare professionals regarding use of ‘nature remedies’, including herbal supplements, might suggest that this is not a major nor essential components in consultations with health care professionals.

Considering the well-established literature on herbal supplements toxicities and interactions with oncology drugs [56], the study finding on limited provider-patient communication on herbal and non-herbal dietary supplements should alarm medical professionals. This is particularly a point of concern in Norway, which in contrast with many Western medical systems, does not implement an integrative oncology service within its oncology centres. This might challenge the communication regarding intake of herbal products and challenge the oncology healthcare professionals educated consultation on the risks versus safety profile of dietary supplements use.

Leaving professional consultations on complementary therapies, dietary and herbal supplements included, to non-medical resources practised outside the oncology centre (e.g., internet, CAM practitioners, friends) is a risk by itself. This practice is in contrast with the integrative oncology literature and the clinical guidelines of the American Society of Clinical Oncology (ASCO) and other leading worldwide oncology societies. This is a very important element of any integrative oncology consultation, which preferable should take place within the oncology centre by physicians and healthcare professionals trained in integrative oncology. Additionally, it should be recorded in the patient’s medical file.

### Implication of the findings

The results of this study can be utilized in several meaningful ways to benefit both patients experiencing cancer and the healthcare system. By leveraging these insights, healthcare professionals, policymakers, and researchers can collectively work towards a more holistic and patient-centred approach to cancer care for men, ultimately improving the overall well-being and quality of life for male cancer survivors.

#### Improving patient support programs

Healthcare organizations and cancer support groups can use these findings to tailor their support programs for men. By understanding the motivations behind male cancer survivors’ implementation of various CAM therapies and dietary changes, more relevant and effective support services can be provided. This might include workshops, counselling, or informational resources focused on the specific CAM methods and dietary changes that experienced to be beneficial for the male cancer survivors.

#### Enhancing healthcare provider education

Oncology professionals can be educated about the widespread use of CAM and dietary supplements among male cancer survivors, and why these modalities are used. This might help better understand their patients’ preferences and potential interactions between conventional treatments and CAM therapies. Additionally, encouraging open communication about CAM usage between patients and healthcare professionals can lead to more comprehensive and safer healthcare practices.

#### Research and integration

The study results provide a basis for further research into the efficacy of specific CAM therapies and dietary interventions. Rigorous scientific studies can be conducted to validate the reported positive outcomes. If certain interventions prove to be consistently beneficial, they can be integrated into standard cancer care protocols.

### Strengths and limitations

This study has several strengths, including a high response rate among male cancer patients, and a panel composition that accurately reflects the general male cancer population in terms of age, cancer site, and geographic distribution across Norway encompassing both rural and urban areas. Notably, the study was conducted outside of a hospital setting, allowing for the inclusion of individuals not currently undergoing conventional cancer treatment.

However, the study should be considered in light of certain limitations. Firstly, with only 153 participants, the study did not achieve the necessary sample size of 384 to represent the entire male Norwegian cancer population. It only adequately represents the male population of the NCS’s user panel, which consists of 218 individuals and required a sample size of 140. The findings of this study are therefore only representative for the user panel of the CSN of which 70.2% of the men responded.

Another limitation of the study is the potential for recall bias as participants may not accurately remember approaches made many years ago. This could result in inaccurate reporting of dietary changes, and the use of special diets, dietary supplements, and CAM.

Information bias could not be ruled out regarding the self-reported nature of the dietary changes, which were not necessarily in line with dietary recommendations [50]. This could have resulted in an overly positive perception of the willingness to make dietary changes as the extent of actual dietary changes was not assessed. The use of predefined answers in the questionnaire might impose the responses and contribute to information bias.

Additionally, the self-observed effect of a self-initiated intervention might be biased in favour of effect according to a wish to justify their untraditional path in cancer treatment. Although all responses were collected anonymously to ensure truthful answers, one cannot completely rule out the presence of social desirability bias [57], which may have caused some participants to exaggerate positive dietary changes and underreported the use of CAM.

## Conclusion

In conclusion, this study sheds light on the coping strategies adopted by male cancer survivors in Norway. The findings underscore the widespread utilization of CAM, dietary supplements, and dietary modifications among male cancer survivors, with a significant majority incorporating these practices into their lives to enhancing immune function and overall quality of life. The high prevalence of CAM and dietary interventions highlights the proactive approach taken by cancer survivors to address the different challenges posed by their condition and its treatments. The incorporation of relaxation techniques and consultations with CAM providers further emphasizes the diverse spectrum of strategies embraced by these individuals. However, it is crucial to note the underreporting of CAM usage and dietary changes to healthcare professionals, suggesting a gap in communication that needs attention. Despite this, the positive health outcomes reported by the participants emphasize the potential benefits of integrating CAM therapies and dietary adjustments into the holistic care of cancer survivors.

## Data Availability

The dataset underlying this paper has not been deposited in any specific repository, all datasets and materials used in this study are, however, available upon reasonable request from corresponding author. Interested parties seeking access to data must be willing to comply with Norwegian privacy regulations, ensuring adherence to stringent data protection standards.

## Abbreviations

CAM: Complementary and Alternative Medicine
NAFKAM: National Research Center in Complementary and Alternative Medicine
NCS: Norwegian Cancer Society
I-CAM-Q: International Questionnaire to Measure Use of Complementary and Alternative Medicine
NSD: Norwegian Centre for Research Data
QoL: Quality of Life
SD: Standard deviation

## References

[1] Cancer in Norway. 2023. Cancer incidence, mortality, survival and prevalence in Norway. In. Edited by Larsen IK. Oslo, Norway: Cancer registry of Norway; 2024.

[2] Strøm MS, Sveen KA, Raknes G, Slungård GF, Reistad SR. Dødsårsaker i Norge 2023 [Causes of death in Norway 2023]. In. Oslo: Norwegian Institute of Public Health; 2024.

[3] Kristoffersen AE, Wider B, Nilsen JV, Bjelland M, Mora DC, Nordberg JH, Broderstad AR, Nakandi K, Stub T. Prevalence of late and long-term effects of cancer (treatment) and use of complementary and alternative medicine in Norway. BMC Complement Med Ther. 2022;22(1):322. 36471296 10.1186/s12906-022-03790-z PMC9721050

[4] Årsrapport. 2022 med resultater og forbedringstiltak fra Nasjonalt kvalitetsregister for prostatakreft [Annual report 2022 with results and improvement measures from the National Quality Register for Prostate Cancer]. In. Oslo, Norway: Cancer registry of Norway; 2023.

[5] Nelson CJ, Kenowitz J. Communication and intimacy-enhancing interventions for men diagnosed with prostate cancer and their partners. J Sex Med. 2013;10:127–32. 23387918 10.1111/jsm.12049 PMC4324570

[6] Ravasco P. Nutrition in cancer patients. J Clin Med. 2019;8(8):1211. 31416154 10.3390/jcm8081211 PMC6723589

[7] Bours MJ, Beijer S, Winkels RM, Van Duijnhoven FJ, Mols F, Breedveld-Peters JJ, Kampman E, Weijenberg MP, Van De Poll-Franse LV. Dietary changes and dietary supplement use, and underlying motives for these habits reported by colorectal cancer survivors of the patient reported outcomes following initial treatment and long-term evaluation of Survivorship (PROFILES) registry. Br J Nutr. 2015;114(2):286–96. 26079602 10.1017/S0007114515001798

[8] Anderson AS, Steele R, Coyle J. Lifestyle issues for colorectal cancer survivors—perceived needs, beliefs and opportunities. Support Care Cancer. 2013;21:35–42. 22773297 10.1007/s00520-012-1487-7

[9] Drotningsvik A. Innspill til Folkehelsemeldingen [Input to the Public Health Notice]. In.; 2022.

[10] Kosthold og kreft [Diet and cancer]. https://kreftforeningen.no/forebygging/kosthold-og-kreft/#:~:text=Et%20sunt%20og%20variert%20kosthold%20kan%20redusere%20risikoen%20for%20kreft,mye%20salt%2 C%20sukker%20og%20fett.

[11] van Zutphen M, van Duijnhoven FJ, Wesselink E, Schrauwen RW, Kouwenhoven EA, van Halteren HK, de Wilt JH, Winkels RM, Kok DE, Boshuizen HC. Identification of lifestyle behaviors associated with recurrence and survival in colorectal cancer patients using random survival forests. Cancers. 2021;13(10):2442. 34069979 10.3390/cancers13102442 PMC8157840

[12] Langlais CS, Graff RE, Van Blarigan EL, Palmer NR, Washington SL, Chan JM, Kenfield SA. Post-diagnostic dietary and lifestyle factors and prostate cancer recurrence, progression, and mortality. Curr Oncol Rep. 2021;23(3):1–20. 10.1007/s11912-021-01017-x PMC7946660 33689041

[13] Clinton SK, Giovannucci EL, Hursting SD. The world cancer research fund/American institute for cancer research third expert report on diet, nutrition, physical activity, and cancer: impact and future directions. J Nutr. 2020;150(4):663–71. 31758189 10.1093/jn/nxz268 PMC7317613

[14] Rock CL, Thomson C, Gansler T, Gapstur SM, McCullough ML, Patel AV, Andrews KS, Bandera EV, Spees CK, Robien K. American Cancer Society guideline for diet and physical activity for cancer prevention. Cancer J Clin. 2020;70(4):245–71. 10.3322/caac.21591 32515498

[15] European Code Against Cancer. 12 ways to reduce your cancer risk. https://cancer-code-europe.iarc.fr/index.php/en/ecac-12-ways/diet-recommendation

[16] Nakandi K, Benebo FO, Hopstock LA, Stub T, Kristoffersen AE. Adherence to lifestyle recommendations among Norwegian cancer survivors and the impact of traditional and complementary medicine use: the Tromsø Study 2015–2016. BMC Complement Med Ther. 2023;23(1):1–12. 10.1186/s12906-023-04123-4 PMC10439550 37598174

[17] Kristoffersen AE, Stub T, Nilsen JV, Nordberg JH, Broderstad AR, Wider B, Bjelland M. Exploring dietary changes and supplement use among Cancer patients in Norway: Prevalence, motivations, Disclosure, Information, and perceived risks and benefits: a cross sectional study. BMC Nutr. 2024;10(1):1–16. 38671478 10.1186/s40795-024-00872-8 PMC11055316

[18] Ford KL, Orsso CE, Kiss N, Johnson SB, Purcell SA, Gagnon A, Laviano A, Prado CM. Dietary choices following a cancer diagnosis: a narrative review. Nutrition 2022:111838. 10.1016/j.nut.2022.111838 36183484

[19] Hamberg K. Gender bias in medicine. Womens Health (Lond). 2008;4(3):237–43. 19072473 10.2217/17455057.4.3.237

[20] Grzymisławska M, Puch EA, Zawada A, Grzymisławski M. Do nutritional behaviors depend on biological sex and cultural gender? Adv Clin Experimental Med 2020;29(1). 10.17219/acem/111817 32017478

[21] Skeie G, Braaten T, Hjartåker A, Lentjes M, Amiano P, Jakszyn P, Pala V, Palanca A, Niekerk E, Verhagen H. Use of dietary supplements in the European prospective investigation into Cancer and Nutrition calibration study. Eur J Clin Nutr. 2009;63(4):S226–38. 19888276 10.1038/ejcn.2009.83

[22] Gardiner P, Sadikova E, Filippelli AC, White LF, Jack BW. Medical reconciliation of dietary supplements: don’t ask, don’t tell. Patient Educ Couns. 2015;98(4):512–7. 25636694 10.1016/j.pec.2014.12.010 PMC4404157

[23] Stub T, Quandt SA, Kristoffersen AE, Jong MC, Arcury TA. Communication and information needs about complementary and alternative medicine: a qualitative study of parents of children with cancer. BMC Complement Med Ther. 2021;21(1):85. 33685422 10.1186/s12906-021-03253-x PMC7938468

[24] Humpel N, Jones SC. Gaining insight into the what, why and where of complementary and alternative medicine use by cancer patients and survivors. Eur J Cancer Care. 2006;15(4):362–8. 10.1111/j.1365-2354.2006.00667.x 16968318

[25] Molassiotis A, Fernadez-Ortega P, Pud D, Ozden G, Scott JA, Panteli V, Margulies A, Browall M, Magri M, Selvekerova S, et al. Use of complementary and alternative medicine in cancer patients: a European survey. Ann Oncol. 2005;16(4):655–63. 15699021 10.1093/annonc/mdi110

[26] Complementary, Medicine A. https://www.cancer.gov/about-cancer/treatment/cam

[27] Lovdata. Act No. 64 of 27 June 2003 relating to the alternative treatment of disease, illness, etc. [http://www.ub.uio.no/ujur/ulovdata/lov-20030627-064-eng.pdf]

[28] Kristoffersen AE, Fonnebo V, Norheim AJ. Use of complementary and alternative medicine among patients: classification criteria determine level of use. J Altern Complement Med. 2008;14(8):911–9. 18990042 10.1089/acm.2008.0127

[29] Kristoffersen AE, Nilsen JV, Stub T, Nordberg JH, Wider B, Mora D, Nakandi K, Bjelland M. Use of complementary and alternative medicine in the context of cancer; prevalence, reasons for use, disclosure, information received, risks and benefits reported by people with cancer in Norway. BMC Complement Med Ther. 2022;22(1):1–21. 35906578 10.1186/s12906-022-03606-0 PMC9336131

[30] Kristoffersen AE, Norheim AJ, Fonnebo VM. Complementary and alternative Medicine Use among Norwegian Cancer survivors: gender-specific Prevalence and associations for Use. Evid Based Complement Alternat Med. 2013;2013:318781. 23606877 10.1155/2013/318781 PMC3625602

[31] Bli med i vårt. brukerpanel [attend our user panel]. https://kreftforeningen.no/engasjer-deg/bli-med-i-vart-brukerpanel/

[32] Quandt SA, Verhoef MJ, Arcury TA, Lewith GT, Steinsbekk A, Kristoffersen AE, Wahner-Roedler DL, Fonnebo V. Development of an international questionnaire to measure use of complementary and alternative medicine (I-CAM-Q). J Altern Complement Med. 2009;15(4):331–9. 19388855 10.1089/acm.2008.0521 PMC3189003

[33] Taherdoost H. Determining sample size; how to calculate survey sample size. Int J Econ Manage Syst 2017:2.

[34] Skeie G, Braaten T, Hjartåker A, Brustad M, Lund E. Cod liver oil, other dietary supplements and survival among cancer patients with solid tumours. Int J Cancer. 2009;125(5):1155–60. 19444919 10.1002/ijc.24422

[35] Tajan M, Vousden KH. Dietary approaches to cancer therapy. Cancer Cell. 2020;37(6):767–85. 32413275 10.1016/j.ccell.2020.04.005

[36] Heine-Bröring RC, Winkels RM, Renkema JM, Kragt L, van Orten‐Luiten ACB, Tigchelaar EF, Chan DS, Norat T, Kampman E. Dietary supplement use and colorectal cancer risk: a systematic review and meta‐analyses of prospective cohort studies. Int J Cancer. 2015;136(10):2388–401. 25335850 10.1002/ijc.29277

[37] Veettil SK, Wong TY, Loo YS, Playdon MC, Lai NM, Giovannucci EL, Chaiyakunapruk N. Role of diet in colorectal cancer incidence: umbrella review of meta-analyses of prospective observational studies. JAMA Netw open. 2021;4(2):e2037341–2037341. 33591366 10.1001/jamanetworkopen.2020.37341 PMC7887658

[38] Lopez-Caleya JF, Ortega-Valín L, Fernández-Villa T, Delgado-Rodríguez M, Martín-Sánchez V, Molina AJ. The role of calcium and vitamin D dietary intake on risk of colorectal cancer: systematic review and meta-analysis of case–control studies. Cancer Causes Control 2022:1–16. 10.1007/s10552-021-01512-3 34708323

[39] Veierød M, Laake P, Thelle D. Dietary fat intake and risk of lung cancer: a prospective study of 51,452 Norwegian men and women. Eur J Cancer Prev. 1997;6(6):540–9. 9496456 10.1097/00008469-199712000-00009

[40] Torfadottir JE, Valdimarsdottir UA, Mucci LA, Kasperzyk JL, Fall K, Tryggvadottir L, Aspelund T, Olafsson O, Harris TB, Jonsson E. Consumption of fish products across the lifespan and prostate cancer risk. PLoS ONE. 2013;8(4):e59799. 23613715 10.1371/journal.pone.0059799 PMC3629172

[41] Zuniga KB, Zhao S, Kenfield SA, Cedars B, Cowan JE, Van Blarigan EL, Broering JM, Carroll PR, Chan JM. Trends in complementary and alternative medicine use among patients with prostate cancer. J Urol. 2019;202(4):689–95. 31091175 10.1097/JU.0000000000000336 PMC7017721

[42] Pouchieu C, Fassier P, Druesne-Pecollo N, Zelek L, Bachmann P, Touillaud M, Bairati I, Hercberg S, Galan P, Cohen P. Dietary supplement use among cancer survivors of the NutriNet-Sante cohort study. Br J Nutr. 2015;113(8):1319–29. 25826598 10.1017/S0007114515000239

[43] Lee HR, Song Y-M, Jeon KH, Cho IY. The Association between the Use of Dietary supplement and psychological status of Cancer survivors in Korea: a cross-sectional study. Korean J Family Med. 2021;42(4):317. 10.4082/kjfm.20.0184 PMC8321910 34320800

[44] Fiskerinasjonen Noreg [The fishing nation Norway]. https://www.regjeringen.no/no/tema/mat-fiske-og-landbruk/fiskeri-og-havbruk/1/fiskeri/fiskerinasjonen/id2577904/

[45] Skåre JU, Brantsæter AL, Frøyland L, Hemre GI, Knutsen HK, Lillegaard ITL, Andreassen ÅK, Elvevoll EO, Andersen LF, Hjeltnes B. Benefit-risk assessment of fish and fish products in the Norwegian diet–an update. Opinion of the Scientific Steering Committee of the Norwegian Scientific Committee for Food Safety. VKM Report; 2014.

[46] Skowronski M, Risør MB, Foss N. Approaching Health in landscapes: an Ethnographic Study with Chronic Cancer patients from a Coastal Village in Northern Norway. Anthropol Action. 2017;24(1):27–33.

[47] Nilsen L, Hopstock LA, Grimsgaard S, Carlsen MH, Lundblad MW. Intake of vegetables, fruits and berries and compliance to five-a-Day in a general Norwegian population—the tromsø study 2015–2016. Nutrients. 2021;13(7):2456. 34371965 10.3390/nu13072456 PMC8308725

[48] Nilsen L, Hopstock LA, Skeie G, Grimsgaard S, Lundblad MW. The Educational Gradient in Intake of Energy and macronutrients in the General Adult and Elderly Population: the Tromsø Study 2015–2016. Nutrients. 2021;13(2):405. 33525333 10.3390/nu13020405 PMC7911135

[49] Kostrådene (Diet recommendations.). https://www.helsedirektoratet.no/faglige-rad/kostradene-og-naeringsstoffer/kostrad-for-befolkningen

[50] Nakandi K, Benebo F, Hopstock LA, Stub T, Kristoffersen AE. Norwegian cancer survivors and adherence to lifestyle recommendations in relation to sex, phase of survivorship, and the use of traditional and complementary medicine in the seventh Tromsø study: a cross sectional study. BMC Complement Altern Med 2023.

[51] Kassianos AP, Raats MM, Gage H, Peacock M. Quality of life and dietary changes among cancer patients: a systematic review. Qual Life Res. 2015;24:705–19. 25218405 10.1007/s11136-014-0802-9

[52] Lin A, Vranceanu A-M, Guanci M, Salgueiro D, Rosand J, Zale EL. Gender differences in longitudinal associations between intimate care, resiliency, and depression among informal caregivers of patients surviving the neuroscience intensive care unit. Neurocrit Care. 2020;32:512–21. 31270671 10.1007/s12028-019-00772-x

[53] Carlson LE, Ursuliak Z, Goodey E, Angen M, Speca M. The effects of a mindfulness meditation-based stress reduction program on mood and symptoms of stress in cancer outpatients: 6-month follow-up. Support Care Cancer. 2001;9:112–23. 11305069 10.1007/s005200000206

[54] Ford CG, Vowles KE, Smith BW, Kinney AY. Mindfulness and meditative movement interventions for men living with cancer: a meta-analysis. Ann Behav Med. 2020;54(5):360–73. 31773148 10.1093/abm/kaz053 PMC7168578

[55] Arends J, Bachmann P, Baracos V, Barthelemy N, Bertz H, Bozzetti F, Fearon K, Hütterer E, Isenring E, Kaasa S. ESPEN guidelines on nutrition in cancer patients. Clin Nutr. 2017;36(1):11–48. 27637832 10.1016/j.clnu.2016.07.015

[56] Ben-Arye E, Samuels N, Goldstein LH, Mutafoglu K, Omran S, Schiff E, Charalambous H, Dweikat T, Ghrayeb I, Bar-Sela G, et al. Potential risks associated with traditional herbal medicine use in cancer care: a study of Middle Eastern oncology health care professionals. Cancer. 2016;122(4):598–610. 26599199 10.1002/cncr.29796

[57] What is Social Desirability Bias?. https://www.scribbr.com/research-bias/social-desirability-bias/

[58] Wma Declaration Of Helsinki. – ethical principles For medical research involving human subjects https://www.wma.net/policies-post/wma-declaration-of-helsinki-ethical-principles-for-medical-research-involving-human-subjects/19886379

